# Immunohistochemistry for Skin Cancers: New Insights into Diagnosis and Treatment of Melanoma

**DOI:** 10.3390/cancers17111769

**Published:** 2025-05-25

**Authors:** Vlad-Mihai Voiculescu, Alina-Ioana Popescu, Mariana Costache

**Affiliations:** 1 Faculty of Medicine, “Carol Davila” University of Medicine and Pharmacy, 050474 Bucharest, Romania; vlad.voiculescu@umfcd.ro (V.-M.V.);; 2 Elias Emergency University Hospital, 011461 Bucharest, Romania; 3 Pathology Department, University Emergency Hospital Bucharest, 050098 Bucharest, Romania

**Keywords:** cutaneous melanoma, immunohistochemistry, skin cancer

## Abstract

**Background**: With its incidence on the rise, a high mortality rate, and great costs associated with its treatment, melanoma represents an important challenge for healthcare systems, clinicians, and pathologists. Therefore, an emphasis should be placed on its early and correct diagnosis, as well as the appropriate assessment of prognostic and predictive factors. Immunohistochemistry (IHC) is an ancillary test that can provide invaluable information for diagnosing melanoma, especially in complex cases. **Objective**: The aim of this systematic review is to gather the available information regarding the use of IHC markers in the diagnosis, differential diagnosis, prognosis, staging, and treatment of melanoma in a format that is easy to access for clinicians and pathologists. **Methods:** A comprehensive search of the literature was conducted and resulted in one hundred and forty-seven studies being included in this systematic review. The results were grouped thematically by specific IHC markers. **Results**: The IHC markers specific to melanocytic differentiation, like S100, SOX10, and Melan-A/MART1, were consistent across studies as being positive in most cases of melanoma, with rare exceptions. HMB-45 and PRAME can provide additional information, especially for differential diagnoses between benign and malignant melanocytic lesions. MITF, Ki67, BRAF, and PD-L1 are associated with prognosis factors, like the Breslow thickness, tumour ulceration, type of inflammatory infiltrate, and response to treatment. **Conclusions**: IHC markers are an invaluable tool for the diagnosis and differential diagnosis of melanoma, especially in cases that lack the characteristic histopathological aspects. In addition, IHC provides prognostic factors and can help in predicting the tumour’s response to various treatments.

## Simple Summary

Melanoma accounts for the majority of skin-cancer related deaths. An early and correct diagnosis is essential for proper patient care. However, melanoma is known to mimic a great number of skin diseases, which makes the diagnosis difficult for both the clinician and the pathologist. Using a proper panel of immunohistochemical markers can greatly improve the diagnostic process. In addition to the diagnostic markers, there are some immunohistochemical markers, like BRAF, PD-L1, and Ki67, that need to be included in the final pathological report because of their predictive and prognostic roles.

## 1. Introduction

Melanoma is defined as a malignant tumour of melanocytes and represents the most aggressive type of skin cancer [1]. The incidence of melanoma has increased over the last several decades, especially in fair-skinned populations. GLOBOCAN 2022 reported an incidence of 331,647 new cases [2]: 7012 cases more than the previous GLOBOCAN report from 2020 [3]. Melanoma also accounts for the majority of skin-cancer-related deaths worldwide, posing a significant public health challenge [2]. Moreover, melanoma also represents a challenge for healthcare systems as the costs of treating patients with melanoma has increased over the years [4]. Considering the aggressive behaviour of melanoma, it is critical to ensure an early and correct diagnosis, as the prognosis declines sharply between the radial and vertical growth phase [5].

Immunohistochemistry (IHC) is an ancillary test used widely in the pathological evaluations of various tissue samples, including skin cancer, in order to detect specific antigens [6]. In clinical practice, IHC is mainly used on formalin-fixed, paraffin-embedded tissues and provides additional, sometimes invaluable, information to the classic haematoxylin–eosin (H&E) examination [7]. This greatly facilitates the differentiation of melanoma from benign nevi and other non-melanocytic tumours [8]. Melanoma is known to mimic a broad spectrum of tumours, both epithelial and mesenchymal. Despite this, there are also the cases of amelanotic or hypomelanotic melanomas which, by definition, lack the presence of melanin pigment, an important clue regarding the tumour’s line of differentiation [9]. Other potential diagnostic pitfalls could be represented by the histopathological variants of melanoma, desmoplastic melanoma, nevoid melanoma, or malignant blue nevus [10]. The use of key immunohistochemical markers, such as S100, SOX10, Melan-A (MART1), HMB-45, and MITF, have demonstrated varying degrees of sensitivity and specificity for the diagnosis of melanoma, assessment of prognostic factors, and guiding of therapeutic decisions [11,12].

Despite its widespread use, the application of IHC in melanomas has some limitations. Technical issues occurring during the staining protocol, the variability of marker immunoreactivity, and interobserver variability when evaluating the slides could impact the diagnostic accuracy [13]. Someof these limitations could be resolved by the use of digital pathology, which has emerged as a transformative approach to the classic analysis of IHC slides. Integratinghigh-resolution scanning and imageanalysis intothe standard interpretation of histopathological slides could increase the consistency of the results across observers and reduce subjectivity [14].

Considering the expanding role of IHC in the diagnosis and therapeutic management of melanoma, a comprehensive evaluation of key immunohistochemical markers, with their strengths and limitations, is warranted. The aim of this systematic review is to synthesisethe existing evidence regarding the use of IHC markers in the diagnosis and treatment of melanoma.

## 2. Materials and Methods

### 2.1. Protocol and Registration

This systematic review was conducted in accordance with the Preferred Reporting Items for Systematic Reviews and Meta-Analyses (PRISMA) guidelines and checklist in Supplementary Materials. The review protocol was not prospectively registered in PROSPERO or any other systematic review registry.

### 2.2. Eligibility Criteria

The inclusion criteria were defined prior to data collection and were as follows:

- Original peer-reviewed research articles, clinical case series, or case reports focusing on the use of immunohistochemistry in melanoma;
- Studies that employed immunohistochemistry for the purpose of the diagnosis, differential diagnosis, prognosis, staging, or treatment of melanoma;
- The inclusion of primary cutaneous melanoma, metastatic melanoma, or histopathological variants such as nodular melanoma, lentigo maligna melanoma, superficial spreading melanoma, amelanotic melanoma, spitzoid melanoma, desmoplastic melanoma, or acral melanoma;
- Articles published in English between January 2000 and May 2025.

The exclusion criteria included the following:

- Animal or in vitro studies;
- Editorials, letters, reviews, or opinion pieces;
- Studies focusing on melanomas in special locations (e.g., mucosal, uveal, ocular);
- Studies not involving immunohistochemistry;
- Articles without the full text available;
- Duplicate publications or secondary analyses from the same dataset.

### 2.3. Information Sources and Search Strategy

A comprehensive search was conducted in two major databases, Pubmed and Scopus. The searches were completed on May 2025. The search strategies combined keywords and subject headings related to melanoma, immunohistochemistry, and relevant clinical applications. Boolean operators (AND, OR) were used to combine the search terms appropriately. Representative search terms included the following:

- “melanoma” AND “immunohistochemistry”.
- “melanoma” AND “S100” OR “SOX10” OR “HMB-45” OR “Melan-A” OR “MART1” OR “KI67” OR “MITF” OR “PRAME” OR “BRAF” OR “p16” OR “PD-L1”.
- “melanoma variants” AND “immunohistochemistry”.

Filters were used to exclude non-human melanomas, in vitro studies, articles not available in English and reviews.

### 2.4. Study Selection

All the identified articles were imported into the Zotero screening database for reference management. Duplicate records were removed. A title and abstract screening was conducted independently by A.I.P. and V.M.V., followed by a full-text screening, to assess eligibility. Discrepancies were resolved by consultation with a third reviewer (M.C.).

**Figure 1:**
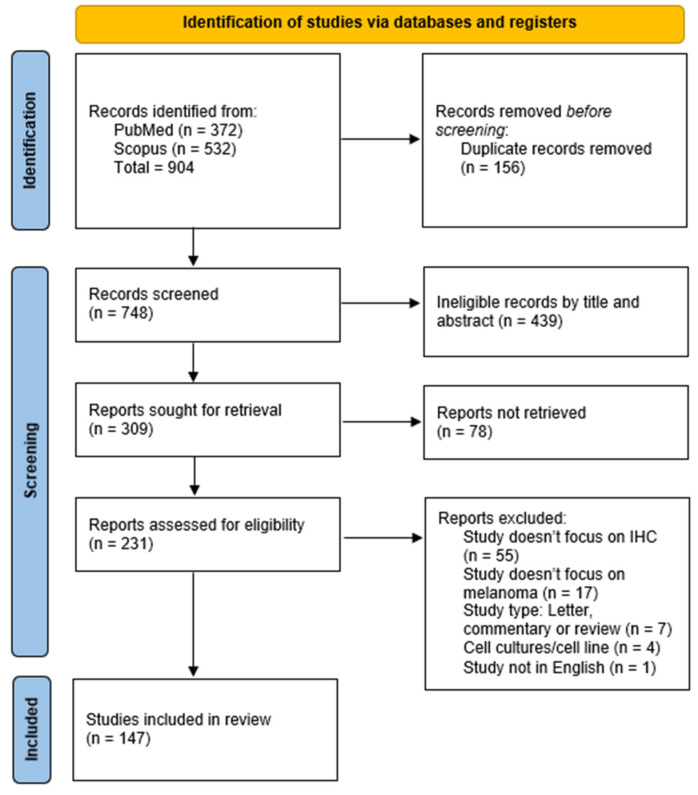
PRISMA 2020 flowchart illustrating the study selection process.

In total, 904 records were identified by the database search, of which, 156 were duplicates. After duplicate removal, 748 records were screened by their title and abstract, out of which, 439 were excluded. Thereafter, 309 reports were sought for retrieval, and 231 reports were retrieved in full and assessed for their eligibility in accordance with the mentioned criteria. Reports were removed for the following reasons: the study does not focus on IHC (55 reports);the study does not focus on melanoma (17 reports);the study type is a letter to the editor, commentary, or review (7 reports); the study is on cell cultures (4 reports); or the study is not in English (1 report). After the full-text assessment, 147 reports were included in the systematic review. The complete selection process is illustrated in the PRISMA 2020 flow diagram (Figure 1).

Additional reports were included through the backward citation of the included articles.

### 2.5. Data Extraction

A standardised data extraction form was used to collect the following information from each included study:

- Melanoma subtype;
- IHC markers assessed;
- Diagnostic, differential diagnostic, staging, prognostic, or treatment applications.

### 2.6. Risk of Bias and Quality Assessment

No formal risk of bias tool was applied because of the heterogeneity of the studies included in terms of their design and reporting.

### 2.7. Data Synthesis

Due to the heterogeneity in the study designs, patient populations, and IHC markers assessed, a qualitative synthesis of findings was performed. The results were grouped thematically by specific IHC markers. No meta-analysis was conducted due to variability in the IHC markers included and the reporting standards.

## 3. Results

### 3.1. S100

**Figure 2:**
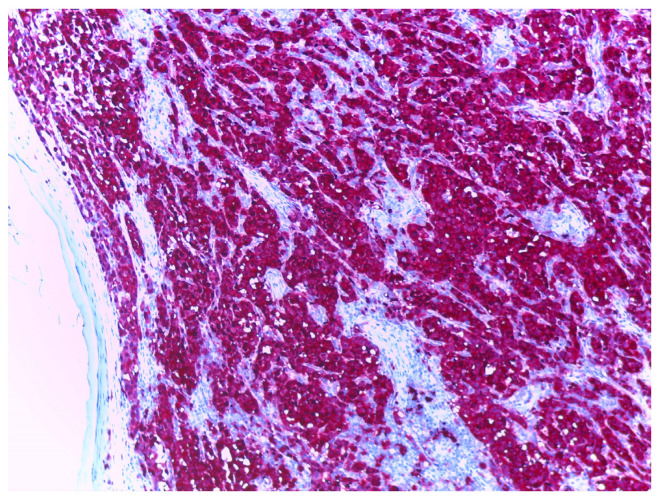
Melanoma stained with S100, at 10× magnification. Nuclei and cytoplasm of melanoma tumour cells are highlighted in red, background stroma highlighted in blue.

S100, particularly the S100B isoform, is a nuclear marker that is broadly used in the diagnosis of primary cutaneous melanoma due to its high sensitivity for cells of melanocytic differentiation [15], with various studies reporting a sensitivity between 90% [11] and 100% for the diagnosis of primary cutaneous melanoma [16,17]. S100 is considered positive when staining is observed both in the cytoplasm and the nucleus (Figure 2).

In routine diagnostic histopathology, S100 is used among other markers of melanocytic differentiation, such as HMB-45 or Melan-A [15]. S100 positivity was reported in primary cutaneous melanomas [18,19,20], Spitzoid melanomas [21], amelanotic melanomas [22,23], nodular melanomas [24], and desmoplastic/spindle cell melanomas [25,26,27,28,29]. There are rare cases reported of S100-negative cutaneous melanomas [30], but more commonly encountered in the literature are the cases of desmoplastic melanoma that donot react with S100 [31]. Immunoreactivity is also maintained in metastases from cutaneous melanomas [32,33,34].

The tumour’s depth of invasion or the Breslow thickness is a critical prognostic marker and a part of the staging process of melanoma. It is measured in millimetres from the top of the granular layer of the epidermis or ulcer base, if present, to the deepest point of the tumour’s penetration and reported to the nearest 0.1 mm [35]. In most cases, the Breslow thickness is assessed on H&E evaluation. Using S100 when measuring the depth of invasion hasdemonstrated greater accuracy than H&E, as well as HMB-45 and Melan-A [36]. Evaluating the presence of lymph node metastases is another important prognostic factor thatis also used in staging melanomas. Lymph node metastases are usually assessed on H&E slides accurately. However, micrometastases are more challenging to detect. One study reported that only 30 to 45% percent of positive lymph nodes were correctly assessed on H&E slides [37], while in another study, 90% of positive lymph nodes were correctly identified with the use of S100 staining [38]. Drawbacks to the micrometastases evaluation by S100 staining include the nuclear expression of the marker, which hide nuclear details of tumoral cells and the reactivity of S100 with other cells from the lymph node, like dendritic cells [39]. The presence of lymphatic or blood vessel invasion is a negative prognostic marker, and its evaluation on H&E has demonstrated a sensitivity of 25% and a specificity of 100% [40]. Using dual staining with S100 and D2-40 demonstrated greater sensitivity(60%) in detecting the lymphatic or vascular invasion [40,41,42,43] factors correlated with a poor prognosis [44]. Other studies recommend pairing S100 with LYVE-1 for the detection of lymphatic or vascular invasion [45].

S100 is not a very specific marker for melanoma as it reacts with various other tumours, such as those derived from nerve sheath tumours, like neurofibromas, Schwannomas, and malignant peripheral nerve sheath tumours (MPNST) [28,46], as well as isolated spindle cells in cutaneous scars. Re-excision specimens occasionally contain rare, isolated, atypical spindle cells demonstrating S100 reactivity, raising the challenge of the differential diagnosis between desmoplastic melanoma and a cutaneous scar. Some authors consider these cells to be Schwann cells [47,48]. In addition, for the diagnosis of spindle cell or desmoplastic melanomas, there are studies that report p75 as being superior to S100 [49].

### 3.2. SOX10

SOX10 is a protein that is positively expressed in melanocytes, Schwann cells, myoepithelial cells, and the luminal cells of sweat glands and salivary glands [50]. In melanocytic lesions, SOX10 is expressed in benign melanocytic nevi [51], primary cutaneous melanomas [19], desmoplastic melanomas [52,53,54], both pure and mixed desmoplastic melanomas [55], and melanoma metastases [56]. SOX10 is highly sensitive and specific todesmoplastic melanoma. Challenges in the differential diagnosis of desmoplastic melanomas include spindle cell squamous cell carcinoma, atypical fibroxanthoma, MPNST, and sarcoma, which were included in a study that reported 100% of desmoplastic melanomas as being SOX10-positive, while all histological mimics were SOX10-negative. Only a few cases of MPNST demonstrated rare, isolated, positive cells [57]. However, others reported up to 49% of MPNSTs as being SOX10-positive, as well as diffuse expression in neurofibromas and Schwannomas [58,59]. There are also reported cases of SOX10-negative desmoplastic melanomas [31]. SOX10 is also expressed in breast carcinoma, which could pose another diagnostic challenge [51].

SOX10 is also expressed in histiocytes from dermal scars, which, in a manner similar to S100, could lead to diagnostic challenges, especially in cases of re-excision specimens. One study evaluated the morphology and immunoreactivity of histiocytes from dermal scars and reported that 71.3% had spindle cell morphology or angulated nuclei, while 86% presented with scattered SOX10-positive cells [60].

Measuring the Breslow thickness using SOX10 has demonstrated greater accuracy than H&E examination and no difference when compared with S100 assessment [36]. Regarding the status of lymph nodes, SOX10 has demonstrated greater accuracy than H&E, S100, Melan-A, and HMB45 in evaluating the presence of micrometastases, as it does not react with other background cells in the lymph node. One drawback of using SOX10 in lymph node sections is the staining of nodal nevi, which could raise a potential diagnostic challenge [61,62].

### 3.3. HMB-45

**Figure 3:**
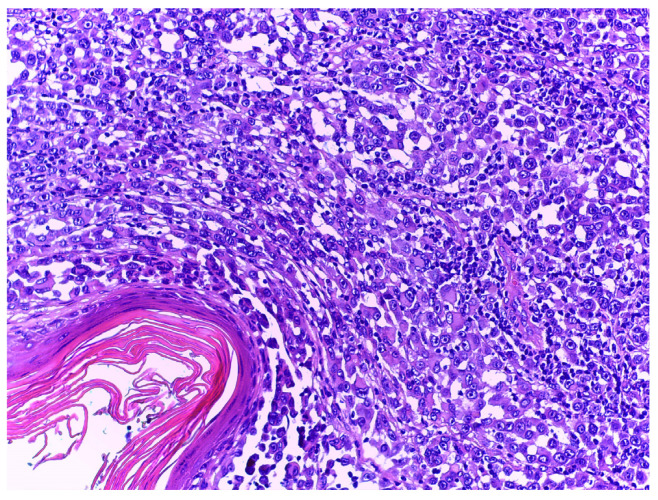
Nodular amelanotic melanoma with H&E stain, at 20× magnification. Tumour cells demonstrate abundant cytoplasm with no melanotic pigment and irregular-shaped nuclei with evident nucleoli.

HMB-45 is a marker of melanocyte differentiation that is less sensitive, but more specific, than S100 for the diagnosis of primary cutaneous melanoma [15], with various studies reporting 56.3 to 77% of melanomas as being HMB-45-positive [11,63,64,65]. Desmoplastic melanomas are usually HMB-45-negative [25,26,27,52,66], with rare cases of positivity [63,67]. HMB-45 is also expressed in amelanotic melanomas (Figure 3) [22,68], including acral amelanotic melanomas [69], primary dermal melanomas [70], and melanoma metastases [63,71,72,73]. Despite being a marker of melanocytic differentiation, the specific pattern of expression allows it to be confidently used in the differential diagnosis between benign and malignant melanocytic lesions. The normal pattern shows HMB-45-positive cells at the junctional level and in the superficial layer of the dermis, while the deeper layers show weak positivity. This gradient is characteristically lost in melanomas, and HMB-45 becomes diffusely expressed [64]. Melanocytic lesions that show HMB-45 expressed in this gradient are more likely to be benign melanocytic lesions than melanomas [74]. HMB-45 is also expressed in blue nevi, Spitz nevi, congenital nevi, dysplastic nevi [64], and deep-penetrating nevi. Nevoid melanomas are usually completely negative in the dermal component [75]. In addition to melanocytic lesions, HMB-45 is also expressed in Schwannomas, angiomyolipomas, lymphangioleiomyomatosis, and clear cell “sugar”tumours [64].

There are various results regarding the use of HMB-45 in evaluating the lymph node status. Some studies report that HMB-45 is not suitable as it also stains other cells from the lymph node [37], while others report 82% percent of micrometastasesbeing diagnosed with HMB-45 [38]. In addition to this, HMB-45 can differentiate between lymph node metastases and nodal nevi [62,76]. Measuring the Breslow thickness using HMB-45 stains demonstrated no difference in accuracy when compared with H&E [36,77].

### 3.4. Melan-A/MART1

Melan-A represents a melanocytic differentiation antigen encoded by the MART-1 gene and normally expressed in mature benign melanocytes. It is also expressed in benign melanocytic nevi and dysplastic nevi [63] with a diffuse and strong staining pattern [15]. Neurotized nevi demonstrated strong and diffuse positivity, in contrast to neurofibromas, whichare usually Melan-A-negative [78]. Primary cutaneous melanomas express Melan-A [79] in 73.3% to 83% of cases [11,63] and is also maintained in melanoma metastases in 71% of cases [63,80]. Desmoplastic melanomas are generally negative for Melan-A [57], with positivity reported in up to 27.3% of cases [67]. Desmoplastic nevi are more frequently positive for Melan-A [81]. Acral amelanotic melanomas also express Melan-A [82]. Some cases of metastatic lesions demonstrated a loss of Melan-Aafter treatment with vemurafenib [83].

Breslow thickness assessments on Melan-A stained slides demonstrated greater accuracy than H&E examination, without exceeding that of S100 [36]. One study reported 59.6% of melanoma biopsies being measured by Melan-A stain as having a greater depth of tumour invasion than was measured by H&E, which led to 33% cases of melanoma in situ to be re-classified as invasive melanoma [84]. Melan-A stain is also preferred in the assessment of the surgical margin during Mohs surgery [85]. It has demonstrated great sensitivity in frozen tissues, with good consistency, decreased background stain, and concordance with H&E margin evaluations on fixed tissue [86]. The lymph node status is accurately assessed by Melan-A stain, with 100% of negative lymph nodes and 98.2% of positive lymph nodes being correctly diagnosed. The percent increased to 100% when additional levels were cut [87], an accuracy level superior to both S100 and HMB-45 [39].

There are several limitations to the use of Melan-A in the primary and differential diagnosis of melanoma. Melan-A could potentially overestimate the number of melanocytes in sun-damaged skin [88]. Melan-A is also positive in “sugar” cell tumours of the lung, PEComa, and angiomyolipoma; however, these tumours usually present with a characteristic morphology that allows a differential diagnosis on H&E staining [89].

### 3.5. Ki67

**Figure 4:**
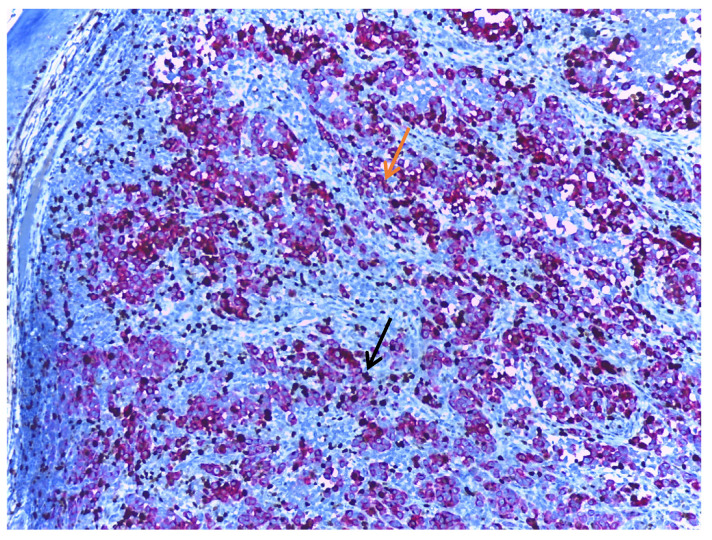
Melanoma dual stained with Melan-A (orange arrow) and Ki67 (black arrow), at 10× magnification. When assessing the Ki67 index, it is recommended to use a dual stain, thus ensuring that the mitotic activity measured is in the tumour cells and not in the adjacent non-tumoral cells. The background stroma is stained blue.

Ki67 is a nuclear-DNA-bindingprotein used as both a diagnostic tool and a prognostic marker in melanoma. Ki67 expression is associated with melanoma progression. Benign melanocytic nevi typically exhibit a lower Ki67-labelling index than primary cutaneous melanomas, reflecting the increased proliferative activity [90,91]. The process of melanoma progression includes the transition from the radial growth phase to the vertical growth phase, a change marked by a shift in the behaviour of tumour cells. In the vertical growth phase, the cells are confined to the epidermis or the papillary dermis, and Ki67 is preferentially expressed in the epidermal component. Shifting to the vertical growth phase is characterised by a higher proliferation capacity and, therefore, a higher Ki67-labelling index [92]. In a similar manner, many studies report that melanoma metastases express a higher Ki67 index than primary tumours [90,93,94]. Also, desmoplastic melanomas express a higher proliferation index than desmoplastic nevi [66]. When assessing the Ki67 index in melanomas, it is recommended to perform a dual stain to ensure that Ki67 is positive in tumour cells (Figure 4).

From a prognostic perspective, higher Ki67 expression in melanoma has been associated with more-aggressive tumourbehaviour and a worse clinical outcome [90,93,95]. Studies have demonstrated that Ki67 expression is associated with the Breslow thickness [90,96]. When comparing the Ki67-labelling index between melanomas in situ and thin melanomas, no differences in proliferation activity were reported. However, metastasizing thin melanomas exhibit higher proliferation activity than non-metastasizing thin melanomas [97]. High Ki67 is also associated with the presence of ulceration, higher mitotic rates, the presence of vascular invasion, tumour necrosis, the Clark level [97], reduced overall survival [98], reduced melanoma-specific survival in acral melanomas [99], and reduced recurrence-free survival [100].

Ki67 assessment has several limitations. Interobserver variability with reduced reproducibility and differences in staining protocols can affect the consistency of results. Most laboratories report the percent of positive cells by eyeballing and using additional techniques, like the manual counting of printed images or automated counting by variousimage analysis software, with the latter greatly improving reproducibility [101].

### 3.6. MITF

MITF, or microphthalmia-associated transcription factor, is encoded by the MITF gene and expressed in melanocytes. In melanomas, MITF is expressed in 56% [65] to 100% of cases when excluding desmoplastic and spindle cells melanomas [102]. MITF seems to be sensitive and specific to epithelioid melanomas, but not for spindle cell and desmoplastic melanomas [103], with studies reporting 1–3% of desmoplastic melanomas as being MITF-positive [102,104]. MITF has exhibited equal or higher sensitivity and specificity than HMB-45 in the diagnosis of desmoplastic melanoma [105].In melanoma metastases, MITF expression is maintained in 23% [65] to 88% of cases [104]. As a marker of melanocytic differentiation, MITF is also expressed in benign melanocytic nevi, with studies reporting up to 83% of cases as positive [65], with similar results for Spitz nevi and dysplastic nevi [102].

MITF expression is associated with a good prognosis [98] and can be used in combination with D2-40 in order to detect lymphatic or vascular invasion [106]. Regarding differential diagnosis, MITF can be used to differentiate actinic keratosis with melanocyte hyperplasia from melanoma in situ with greater efficacy than SOX10 [107]. When evaluating melanocytes in sun-damaged skin, MITF melanocyte counting is more accurate than Melan-A, which tends to overestimate the melanocyte number. This derives from the nuclear expression of MITF compared with the cytoplasmic expression of Melan-A. Additionally, MITF can be used to evaluate the pagetoid spread [88].

### 3.7. PRAME

**Figure 5:**
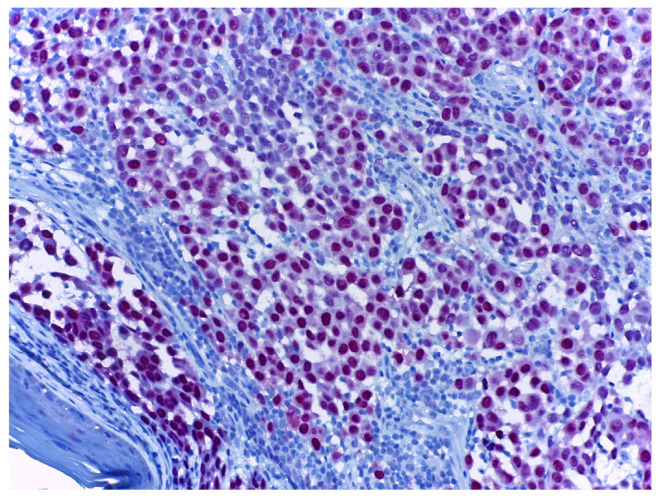
Melanoma stained with PRAME (red), at 20× magnification. Melanoma tumour cells with red-stained nuclei demonstrate PRAME positivity.

PRAME, or Preferentially-Expressed Antigen in Melanoma, is an IHC marker that is mainly used in the differential diagnosis of benign and malignant melanocytic lesions [108,109]. Positive staining is considered nuclear (Figure 5). Melanomas are traditionally diffusely positive for PRAME, which is defined as expression in >75% of tumour cells. Studies report various percentages of positivity for PRAME, from 59.1% up to 89.6% [64,110,111,112,113]. Its sensitivityhas been reported as 73.6% and its specificity as 96.5% [64]. Superficial spreading melanomas and nodular and lentigo maligna melanomas also express PRAME in 88.2–90.9% of cases [111]. Also, acral and subungual melanomas exhibit PRAME positivity [114,115]. Its sensitivity and specificity for subungual melanomas have been reported as 76.9% and 92.9%, respectively [116]. Spindle cell and desmoplastic melanomas are more frequently negative for PRAME staining, and in cases of mixed desmoplastic melanomas, staining is usually positive in the non-desmoplastic component [117]. There are rare cases of PRAME-negative melanomas, predominantly those with spindle-cell morphology [64,118]. PRAME is also expressed in melanomain situ cases [111] and can aid the early diagnosis of melanoma [119].

When compared with S100 and SOX10, PRAME was demonstrated to be less sensitive but much more specific because of its limited expression in benign melanocytic lesions. Benign melanocytic nevi are usually negative for PRAME [64,120], with rare cases reported of scattered positive cells, especially at the junctional layer [121]. Spitz nevi, dysplastic nevi, and Reed nevi are also predominantly negative for PRAME, with rare cases of scattered positive cells [118]. In cases of melanoma developing on benign nevi, PRAME demonstrated positivity only in the melanoma component [122]. Metastases of primary cutaneous melanomas also exhibit PRAME expression [90]. Lymph node metastases exhibit PRAME in >50% of tumour cells, while nodal nevi can also exhibit PRAME positivity, usually in <50% of cells. In these cases, studies recommend pairing PRAME with p16 as the latter stains nodal nevi in >50% of cells [123]. One study reported a negative prognosis in correlation with PRAME staining [124].

Regarding differential diagnosis, PRAME can also differentiate between solar lentigo, non-lesional sun-damaged skin with melanocyte hyperplasia [125], melanocytic pseudonests in lichenoid reactions, and melanoma in situ [126]. PRAME results are in 90% concordance with FISH for melanoma and SNP-array [127].

This highly specific expression profile makes PRAME a valuable IHC stain in the histopathological examination of surgical margins [128], as normal adjacent or inflamed skin does not react with PRAME. This technique demonstrated equal, if not superior, results when compared with H&E assessment [117,129].

The limitations of PRAME use include positive expression in various other tumours, such as synovial sarcoma, myxoid liposarcoma, malignant peripheral nerve sheath tumours, breast cancer, renal cell carcinoma, and ovarian carcinoma [108].

### 3.8. BRAF

Around 50% of primary cutaneous melanomas show BRAF mutations, and out of these, 90% are a distinct mutant variant that forms by the replacement of valine by glutamic acid on exon 15 of the BRAF gene, named the BRAF V600E mutant variant [130]. The presence of the BRAF V600E mutant variant in melanoma is associated with several clinical characteristics. These mutations are more frequently encountered in melanomas that develop on the trunk, rather than the head and neck regions, or other sun-protected sites. This supports the theory of intermittent sun exposure in the pathophysiology of melanoma. Additionally, melanomas that develop on skin surfaces with signs of cumulative sun damage, like marked solar elastosis, show a low frequency of BRAF V600E mutations [131], but these cases show the less commonBRAF V600K mutation. This is characterised by the replacement of valine with lysine [132]. The BRAF V600K mutant melanoma is more frequently associated with older age, male gender [133], and scalp location [134] and have demonstrated more-aggressive behaviour than BRAF V600E, with an inferior response to treatment and shorter progression-free survival after a combination of BRAF and MEK inhibitors [135]. Melanomas harbouring BRAF V600E mutations tend to develop in younger patients, compared to the BRAF wild-type [136]. The BRAF V600E mutation is also associated with sentinel lymph node metastasis [137] but not with ulceration or the host immune response measured by TILs, as reported by some of the included studies. [138]. Spitzoid melanomas with BRAF mutations are also associated with worse clinical outcomes [139].

Other studies report that positive BRAF V600E expression is associated with the tumour thickness, the presence of ulceration, reduced overall survival [140], and non-brisk inflammation [141]. BRAF V600E mutations have also been reported in nodal nevi from patients with stage II melanoma. Out of these, 33% of patients also had positive lymph nodes in the same stations as the nodal nevi [76]. Metastatic melanomas usually present the same mutant variant as the primary tumour, with rare cases of discordance [142].

The presence of a BRAF mutation can confidently be assessed by IHC, having demonstrated comparable results with PCR and pyrosequencing [143]. One drawback to using IHC is the use of VE1 antibody that only recognises the BRAF V600E mutant variant [144,145].

BRAF V600E mutation acts as predictive marker for the response to specific treatments. Primary cutaneous melanomas that harbourthe mutation can respond to inhibitor treatments [146], like BRAF inhibitors, which include vemurafenib, dabrafenib, and encorafenib, and MEK inhibitors, like trametinib, binimetinib, and cobimetinib [147].

### 3.9. P16

P16 expression in primary cutaneous melanomas has exhibited variable behaviour, with some studies reporting all melanomas as being p16-negative [148], while others reported up to 72% positivity [149]. Because of this variable expression, p16 is more often used as an adjunct tool in challenging cases where histopathologic features do not clearly distinguish benign nevi from malignant melanoma. Benign nevi [112,150], as well as Spitz nevi [151,152] and congenital nevi, typically show moderate or strong staining for p16 [150,153]. In contrast, primary melanomas and metastatic lesions demonstrated a reductionor loss of p16 expression in a variety of cases, with exceptions as mentioned above [154]. Different expression patterns havealso been observed between the radial and vertical growth phases of melanoma [155], withstrong p16 expression in microinvasive dermal nests, marking the transition between the two phases [156]. Acral lentiginous melanomas retain p16 expression [157].

The loss of p16 expression is associated with a worse prognosis, reduced overall survival [158], vascular invasion, lymph node metastases, the recurrence of melanoma, metastases [159], and reduced inflammatory infiltrates in thetumoral microenvironment [160]. Regarding the Breslow thickness, the results are variable, with studies reporting an association between the loss of p16 and a greater depth of invasion [161], while others report no association [162]. In thin melanomas, p16 loss is associated with relapsing [163].

### 3.10. PD-L1

Programmed death-ligand 1 (PD-L1) is expressed on the surface of melanoma tumour cells and tumour-infiltrating immune cells. It binds to the PD-1 receptor on activated T-cells, leading to the suppression of the cytotoxic immune response. PD-L1 expression on tumour cells has been recognised as a negative prognostic marker as it correlates with the shorter 5-year survival rate, the Clark level, the presence of lymph node metastases [164], the mitotic rate, the Ki67 index, metastases, the presence of ulceration [165], and perineural invasion [166]. Other studies reported that PD-L1 expression in tumour cells is correlated with higher TIL infiltration in the tumour microenvironment [167]. Most cases of melanomas with PD-L1-positive expression develop on chronically sun-damaged skin [168]. Nodular-type melanomas are more frequently PD-L1-positive than superficial spreading, acral lentiginous, and lentigo malignamelanomas, even when adjusted for the tumour thickness [169]. Lymph node micrometastases express PD-L1 [170].

PD-L1 expression is an important marker in evaluating melanomas in the context of predicting their response to immune checkpoint inhibitors [171], like nivolumab or ipilimumab [172]. PD-L1 overexpression is associated with a better response to therapy [173]. PD-L1 expression is not the sole determinant of the immunotherapy response, and its level of expression is also affected by prior therapies and the presence of TILs. However, PD-L1 is still considered as a predictive marker in melanoma [174].

## 4. Conclusions

Melanoma represents a challenge from both the clinical and pathological point of view. With a high incidence that continues to increase, a high mortality rate, and enormous costs associated with treatment, this melanocytic malignancy is also a challenge for healthcare systems worldwide [2,4]. Thus, an emphasis must be placed on its early and correct diagnosis in order to improve patient’s prognosis. Melanoma is known as a great mimicker. From the clinician perspective, amelanotic or hypomelanotic types of melanomas could pose a significant challenge and delay a correct diagnosis and treatment. When evaluating the morphological characteristics, the list of possible lesions that could resemble melanoma and, thus, enter the differential diagnosis list is vast, especially when considering all the histologic variants of melanoma [9]. An especially difficult differential diagnosis for the pathologist is that between benign, atypical, and malignant melanocytic lesions, as the evolution, prognosis, and treatment of these entities differ considerably. Additionally, when confronted with a case of melanoma, it is not only important to correctly diagnose the case but also to include in the report all the prognostic and predictive factors [12]. This allows a clear view of the case, the prognosis of the patient, and the possible lines of treatment. Measuring the Breslow thickness; assessing vascular and perineural invasion; measuring and reporting surgical margins;and reporting the BRAF status, mitotic activity, presence or absence of ulceration, and the Clark level of invasion are all factors that need to be included in the final report of a melanoma diagnosis. All of these could potentially be assessed by classic H&E examination, but with greater interobserver variability, some factors, like perineural or vascular invasion, could be missed [175].

The potential differential diagnosis challenges and the assessment of prognostic and predictive factors could be greatly improved by using IHC staining. Using markers of melanocytic differentiation can establish the tumour’s cell line of differentiation, thus shortening the list of possible diagnoses. These are represented by S100, SOX10, and Melan-A/MART1 [37,176]. S100 is considered the most sensitive marker for melanoma, but its specificity is low, which limits its use as a singular marker. It is recommended, however, when paired with another IHC marker, usually HMB-45, which has demonstrated great specificity [176]. HMB-45 and PRAME are IHC markers to use when faced with a difficult diagnosis between benign and malignant melanocytic lesions. PRAME is expressed almost exclusively in malignant melanocytic lesions, while for HMB-45, the difference is made by its unique pattern of expression. The Ki67 proliferative index must be included in the final report, being a prognostic factor [94,177]. In the same manner, BRAF and PD-L1 must also be included in the report to establish the possible line of treatment, theirroles as predictive markers having been established in a great number of studies [168,178].

The list of IHC markers included in this review and their roles are summarised in Table 1.

Additional markers could be included in the IHC panel depending on the specific challenge the pathologist faces. MITF could provide important information when faced with the differential diagnosis of actinic keratosis with melanocyte hyperplasia and melanoma in situ, while p16 could help the diagnosis of a primary tumour and provides additional prognostic information [88,162].

The limitations of this systematic review include the heterogeneity of the study designs and methodologies, which make direct comparison difficult;a publication bias;the variability of IHC interpretation (manual vs. digital), which may affect the reproducibility and the advances in IHC technology over time, which may affect the relevance of the older studies included. In this context, performing a subgroup analysis based on methodological differences or more clearly defined inclusion criteria could ensure only the inclusion of studies reporting standardised scoring systems.

In conclusion, this systematic review proposes a comprehensive list of IHC markers for diagnosing melanoma and expands on their roles in the primary diagnosis, differential diagnosis, and assessment of prognostic and predictive factors. Future efforts may include focusing on studies with standardised scoring systems, which may also make it possible to conduct a meta-analysis.

## Figures and Tables

**Table 1:** The IHC markers included in the systematic reviews and the main findings for each.

IHC Marker	Main Findings
S100	Positive in most melanoma cases and histologic subtypes. Superior to H&E, Melan-A, and HMB-45 in measuring Breslow thickness.
SOX10	Highly sensitive and specific to the diagnosis of desmoplastic melanoma. Superior to H&E in measuring the Breslow thickness. Superior to H&E, S100, HMB-45, and Melan-A in evaluating lymph node micrometastases.
HMB45	Diffuse staining with a loss of gradient in melanomas, compared with melanocytic nevi. Differentiates between lymph node metastases and nodal nevi.
Melan-A	Superior to H&E in measuring the Breslow thickness. Suitable for the assessment of the surgical margin status in Mohs surgery; great concordance with paraffin-fixed tissue examination.
Ki67	A prognostic marker associated with the presence of ulceration, higher mitotic rates, the presence of vascular invasion, tumour necrosis, the Clark level, reduced overall survival, reduced recurrence-free survival.
MITF	Higher sensitivity and specificity than HMB-45 in diagnosing desmoplastic melanoma. A good prognosis marker.
PRAME	Suitable for differential diagnosis between benign and malignant melanocytic lesions.
BRAF	A prognostic marker associated with the tumour thickness, presence of ulceration, reduced overall survival, and non-brisk inflammation. Predicts the tumour response to BRAF inhibitors.
P16	A loss of expression is associated with a worse prognosis, reduced overall survival, vascular invasion, lymph node metastases, the recurrence of melanoma, metastases, and reduced inflammatory infiltrates in the tumoral microenvironment.
PD-L1	A prognosis marker associated witha shorter 5-year survival rate, the Clark level, the presence of lymph node metastases, the mitotic rate, the Ki67 index, metastases, the presence of ulceration, and perineural invasion. Predicts thetumour response to immune checkpoint inhibitors.

## Data Availability

No new data were created or analysed in this study. Data sharing is not applicable to this article.

## Supplementary Materials

The following are available online at https://www.mdpi.com/article/10.3390/cancers17111769/s1, PRISMA 2020 checklist. Ref. [179] is cited in Supplementary Materials.
